# Home environment conditions during childhood and psychosocial outcomes across three generations in Sweden: population based adoption-discordant sibling comparison study

**DOI:** 10.1136/bmj-2025-087844

**Published:** 2026-04-22

**Authors:** Zhenxin Liao, Mengping Zhou, Isabell Brikell, Zheng Chang, Ralf Kuja-Halkola, Brian M D’Onofrio, Henrik Larsson, Paul Lichtenstein, Erik Pettersson

**Affiliations:** 1 Department of Medical Epidemiology and Biostatistics, Karolinska Institutet, Stockholm, Sweden; 2 Beijing International Center for Mathematical Research, Peking University, Beijing, China; 3 Chongqing Taiji Industry, Sinopharm, Chonginq, China; 4 Department of Psychological and Brain Sciences, Indiana University, Bloomington, IN, USA; 5 School of Medical Sciences, Örebro University, Örebro, Sweden

## Abstract

**Objectives:**

To examine if early adoption into a family with favourable home environment conditions reduces long term psychosocial risks and provides intergenerational benefits among individuals born to parents with psychiatric or behavioural problems.

**Design:**

Population based adoption-discordant sibling comparison study.

**Setting:**

Swedish registers of births between 1950 and 1980, with follow-up to 31 December 2020.

**Participants:**

Two samples of sibling pairs from at risk families: 4254 full siblings and 7796 maternal half siblings, who were or were not adopted before age 10 years. Offspring of these siblings (born 1965-2020) were also followed to assess intergenerational spillover effects.

**Main outcome measures:**

The variable of interest was adoption into non-biological families. Outcomes included psychiatric diagnoses, long term unemployment, receipt of social welfare, highest attained education (and upper secondary education ineligibility for generation 3), criminal convictions, and, among men, non-cognitive skills and general intelligence assessed at military conscription.

**Results:**

Adopted individuals (n=1535) showed a lower risk of psychiatric disorders (29.8% *v* 36.1% among non‑adopted siblings), criminal convictions (26.1% *v* 34.0%), and receiving social welfare (37.8% *v* 48.5%). They also showed higher mean intelligence scores (4.5 *v* 3.8) and non-cognitive skills scores (4.8 *v* 3.9) and were more likely to have attended university (26.0% *v* 15.2%). The offspring of adopted individuals (n=2750), in turn, also displayed modestly higher psychosocial functioning than their cousins (eg, 29.6% *v* 32.3% with psychiatric disorders), although the associations were weaker and less precisely estimated. This pattern of associations was similar in the maternal half sibling sample.

**Conclusions:**

Early adoption into a family with favourable home environment conditions was associated with moderate, enduring benefits across psychiatric, social, and cognitive outcomes, extending into the next generation. These results highlight the potential for environmental improvements during childhood to mitigate intergenerational disadvantage.

## Introduction

Children raised in suboptimal home environments have an increased risk of mental health problems, lower educational attainment, and worse performance in the labour market.^1^ ^2^ ^3^ ^4^ Although societies have invested in improving the environments for children in at risk families to counter this risk, debate remains as to the effectiveness of such interventions.^5^ ^6^ ^7^ ^8^ ^9^ ^10^

Three high quality, high cost randomised clinical trials in preschool aged children observed a protective effect of improved rearing conditions. In the Perry Preschool Project (n=123), which targeted American children aged 3-4 years from disadvantaged families in the 1960s, those randomised to an intervention for two years (including 12 hours of training each week aimed at foster social skills and self-control, in addition to two hour weekly home visits), were rated as having fewer externalising problems at ages 7-9 years.^11^ The Abecedarian Project (n=111), which randomly assigned a similar intervention to at risk children aged 0-5 years, also showed improvements in psychosocial outcomes.^12^ The more recent multisite Infant Health and Development Program (n=985), which was modelled after the Perry and Abecedarian projects, likewise generated protective effects up to age 18 years.^13^ ^14^ The evidence from the lower cost Head Start programme, which provides education to young children in low income families in the US (modal enrolment duration one year), is somewhat mixed but still seems to favour an enriched childhood environment as being protective.^5^ ^15^ A handful of studies also indicate that the beneficial effects of improved child-rearing conditions spill over to the third generation (ie, to offspring of the treated children).^16^ For instance, the grandchildren of tribe members of a southeastern native American Indian community who received annual cash transfers, displayed higher test scores in third grade (compared with grandchildren of non-recipient tribe members from the same community).^17^ Similarly, offspring of individuals from the Perry and Abecedarian studies who were randomised to the early childhood interventions were more likely to graduate from college, be employed, and be in good physical health.^18^

Despite those studies, the literature on early childhood interventions is far from uniform, and researchers have questioned whether improved child-rearing conditions boost functioning in the long term.^6^ ^19^ This scepticism stems from recent evaluations of large scale programmes (eg, Tennessee’s Voluntary Pre-K) and relatively low intensity interventions such as income transfer experiments, both of which have reported null or even adverse effects on early outcomes.^9^ ^20^ Furthermore, evidence indicates that short term improvements in achievement test scores tend to diminish (fade-out) after completion of the programme^10^ ^21^ ^22^ ^23^; however, fade-out in test scores might not necessarily imply an absence of long term benefits, as some research suggests that early childhood interventions can yield durable improvements in outcomes in adulthood, including educational attainment, health, and earnings, even when earlier cognitive gains diminished.^15^ ^17^ ^24^ ^25^ ^26^ ^27^ Another source of scepticism stems from a meta-analysis of early intervention programmes in the US, which observed decreased effect sizes over time, presumably as a result of societies extending their safety nets such that the gap between treatment and control groups might have decreased over time.

In two samples of siblings born in Sweden to parents with psychiatric or behavioural problems, we examined if improved home environment conditions through early adoption reduced long term psychosocial risks and provided intergenerational benefits.

## Methods

### Study design

Using unique personal identification numbers, we conducted a population based cohort study, linking several Swedish national registers: the Total Population Register, Multi-Generation Register, National Patient Register, the National Crime Register, Cause of Death Register, Population and Housing Census, Longitudinal Integration Database for Health Insurance and Market Studies, Swedish Military Conscription Register, and National Medical Birth Register. Together, these registers provide comprehensive information on personal characteristics, family relationships (including biological and adoptive links), psychiatric diagnoses, cognitive and non-cognitive skills (among male conscripts), criminal convictions, educational outcomes, and performance in the labour market.

The supplementary file provides a detailed history of national adoptions in Sweden. Briefly, adoption in Sweden became legally regulated in 1917, with 3000 children adopted annually around 1950, which thereafter declined, largely as a result of the expansion of social welfare, increased access to birth control, and decreasing stigma about single motherhood.^28^ The biological mothers were disproportionately young, unmarried, and from lower socioeconomic backgrounds, whereas the adoptive parents were typically older, married, childless, and from higher socioeconomic backgrounds.^29^ ^30^ Extensive historical reviews found that the majority of children were placed into adoptive families during early childhood (mostly before age 2 years), and that the main reason for adoption was a lack of resources.^29^ ^31^ ^32^

### Participants

**Fig 1:**
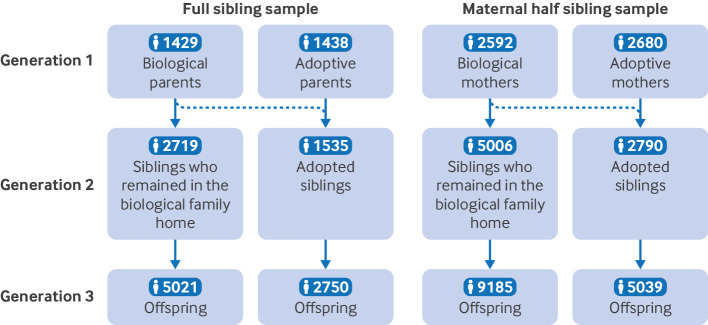
Definition of three generations. In Generation 1, at least one biological parent had a history of psychiatric disorder, suicide (attempt or death), or criminal behaviour. Solid arrows denote genetic and rearing status, dotted arrows denote rearing only status (adoptive parents), and dashed arrows denote genetic only status (biological parents did not rear the child). The numbers of biological and adoptive parents are not identical because some biological families had multiple children adopted, often into different families, and a small number of adoptive families received children from more than one biological family (see supplementary file for more information)

Using the Swedish Multi-Generation Register, we identified two large samples of siblings (one with full siblings and one with maternal half siblings), both of which spanned three generations (fig 1) and were discordant for adoption before age 10 years, and where the biological parent or parents had a history of a psychiatric diagnosis, suicide (attempt or death), or criminal behaviour (court convictions or suspicion of a violent or property crime).^33^ ^34^ The adoptive parents were carefully screened for suitability, and as such served as a proxy for improved child-rearing conditions.^29^ ^30^ ^31^ ^32^ We then examined the extent to which the adopted siblings displayed improved functioning in adulthood compared with their siblings who had remained in the biological family home, and if these improvements transferred to the next generation.

### Biological parents (generation 1)

In line with previous studies,^33^ ^34^ for generation 1 in the full sibling sample, we identified at least one biological parent who had a lifetime history of a psychiatric diagnosis, suicide (attempt or death), or criminal behaviour (court convictions or suspicion of a violent or property crime), and who had at least one child adopted and at least one remaining in the biological home, both before age 10 years (n=1429 pairs of biological parents). We used the housing unit identifiers from the Population and Housing Census^35^ (based on the 1960, 1965, 1970, 1975, 1980, 1985, and 1990 censuses) to identify whether siblings lived in the same building as their biological or adoptive parents. We applied the same selection criteria to the maternal half sibling sample (n=2592 biological mothers).

### Generation 2 (adopted or remained in the biological home)

Generation 2 included generation 1’s offspring who remained in the biological home or were adopted (n=4254 full siblings; n=7796 maternal half siblings, born 1950-80). We excluded adopted children who later returned to live with their biological parents or were adopted by uncles or aunts. To estimate the timing of adoption, we constructed a proxy measure defined as the child’s age at the first census wave in which co-residence with at least one adoptive parent was observed. Because the Population and Housing Census was conducted at five year intervals, this measure represents the upper bound of the true age at adoption, and the actual transition into the adoptive household is likely to have occurred earlier. The mean age at first observed co-residence with adoptive parents was 3.6 years in the full sibling sample and 4.1 years in the maternal half sibling sample.

### Generation 3 (offspring of generation 2)

Generation 3 included generation 2’s offspring (n=7771 full siblings, born 1965-2020; n=14224 maternal half siblings, born 1966-2020).

### Variable and outcomes of interest

The variable of interest was whether siblings (ie, generation 2) were raised by the biological parents or biological mother versus the adoptive parents.

We identified the same psychiatric, labour market, educational, and behavioural outcomes in generations 2 and 3 by linking several national population based registers (see supplementary table A for data sources and supplementary table B for definitions of outcomes). The end of follow-up was 31 December 2020.

*Psychiatric outcome*—we defined this outcome as the first record of any lifetime psychiatric diagnosis, without differentiating specific diagnoses according to ICD (international classification of diseases, eighth, ninth, and 10th revisions) codes 290-319, 290-319, and F00-F99, respectively.

*Labour market outcomes—*these included the first occurrence of long term unemployment, defined as ≥180 consecutive days of registered unemployment in each calendar year, and the first receipt of social welfare after age 20 years.

*Educational outcome*—we included the highest attained level of education (recorded between 1990 and 2020), measured as a continuous numerical variable ranging from 1 to 7 (1=primary or lower secondary education for <9 years, 2=primary or lower secondary education for 9 years, 3=upper secondary education for ≤2 years, 4=upper secondary education for 3 years, 5=post-secondary education for <3 years, 6=post-secondary education for ≥3 years, and 7=postgraduate education). Additionally, for generation 3, we included eligibility or ineligibility for upper secondary education, which was determined upon completion of year 9 when students are aged 15-16 years. Ineligibility resulted from failure to pass core subjects (nationally, about 10-15% of such students each year).^36^

*Behavioural outcomes*—these included the first occurrence of a court conviction for a violent or property crime, non-cognitive skills, and general intelligence. The last two were assessed among male conscripts during mandatory military service at age 18 years, when trained psychologists evaluated non-cognitive skills (emotional stability, leadership potential, and suitability for military service) during a 20-30 minute semistructured interview^37^; and general intelligence was measured with a battery of written tests covering verbal, spatial, logical, and technical abilities (mean completion time was 62 minutes), aggregated into a general cognitive ability score.^38^ Both cognitive and non-cognitive skills were transformed into stanine scores.^1^ ^2^ ^3^ ^4^ ^5^ ^6^ ^7^ ^8^ ^9^ The interrater reliability of the non-cognitive score was estimated at 0.85 based on 30 taped interviews, and the internal consistency reliability of the cognitive test was 0.90.^37^ ^39^ ^40^ The cognitive test predicts education level over and above parental socioeconomic status,^41^ and the non-cognitive test predicts psychiatric diagnoses decades later,^42^ supporting the validity of the tests. These data are available for more than 90% of Swedish men born between 1951 and 1988 and tested between 1969 and 2006.^43^

### Missing data

Although the registers have complete coverage, data were missing on four outcome variables due to sex and starting dates. Measures of non-cognitive skills and general intelligence were only available for male conscripts born between 1951 and 1988.

Information on highest educational attainment was obtained from the Longitudinal Integration Database for Health Insurance and Market Studies. Although this register started in 1990, it includes previous highest education recorded through the Education Register. Nevertheless, as some generation 1 individuals were born more than a century ago, their highest education level was missing.^44^

Eligibility for upper secondary education was obtained from the Swedish National School Register, available for cohorts born from 1982 onwards.

### Covariates

To control for potential cohort effects, we included generation 2’s birth year as a covariate. Sex was not assumed a priori to be a confounder of the adoption-outcome associations, as previous adoption studies have not identified the sex of a child to be associated with adoption placement^45^ ^46^ ^47^; nevertheless, we included it in a sensitivity analysis.

### Statistical analysis

To control for unmeasured familial factors, we analysed associations within family clusters.^48^ To account for non-independent observations, we estimated robust (sandwich) standard errors.

Stratified Cox proportional hazard regression models examined associations between adoption status and the time-to-event outcomes (incidence rate reported in supplementary tables V-Y),^49^ including any psychiatric diagnosis, long term unemployment, criminal behaviour, and receipt of social welfare. Time-to-event outcomes were calculated as the number of days from birth to the earliest event date, death, migration, or end of follow-up (31 December 2020), and was converted to age at event as the time scale. Individuals who did not experience the event were censored at the earliest of death, migration, or end of follow-up. For the binary outcome of eligibility or ineligibility for upper secondary school, we fitted a fixed effects logistic regression model, and for the continuous outcomes (non-cognitive skills, general intelligence, and highest education), we fitted fixed effects linear regressions (for highest education, we first excluded those who died or emigrated before age 30 years).

Data was managed using SAS version 9.4. Data analyses were performed using R version 4.3.1 with the packages stats,^50^ survival,^51^ and fixest.^52^ ^53^ To account for multiple hypothesis testing, the P value threshold was adjusted using the Benjamini-Hochberg procedure to control the false discovery rate at 0.05 (for 30 tests).^54^

#### Sensitivity analyses

To evaluate the robustness of our findings and assess potential sources of bias, we conducted six sensitivity analyses. Firstly, to explore potential sex specific effects, we performed sex stratified analyses in generations 2 and 3. In addition, we re-ran the main within sibling models for generation 2 with sex included as an additional covariate.

Secondly, to assess whether historical changes in adoption practices might have influenced the results, we conducted cohort stratified analyses in generation 2 by birth periods (1950-65 and 1965-80). In addition, to address differences in age and right censoring in generation 3, we performed cohort stratified analyses by offspring birth periods (1965-80, 1980-95, and 1995-2010).

Thirdly, to examine if unique child characteristics might be associated with adoption status, we conducted two sensitivity analyses. We analysed a subsample restricted to biological mothers who gave birth before age 21 years and had their firstborn child adopted, such that adoption was likely attributable to external circumstances; and we examined the association between small-for-gestational-age status (available in a subsample born 1973-80) and adoption status using within family logistic regression models with family fixed effects to directly assess if observable perinatal health predicted adoption.

Fourthly, to characterise potential differences in selection patterns, we examined the distribution of birth order among adopted and unadopted individuals in the full sibling and maternal half sibling samples.

Fifthly, to reduce potential heterogeneity related to later placements, we restricted the sample to individuals who were relocated to their adoptive parents before age 5 years in both the full sibling sample and the maternal half sibling sample.

Finally, to examine comparability and generalisability across different types of families, in generation 1 we examined the prevalence of different types of psychiatric diagnoses across biological and adoptive parents and examined socioeconomic characteristics across families with all versus none of their children adopted.

### Patient and public involvement

No patients or members of the public were directly involved in the design, conduct, or analysis of this registry based study. The study was based on pseudonymised population registers, which precluded direct involvement of the individuals included in the data. In developing the research questions and interpreting the findings, however, we reviewed existing public discourse, policy debates, and previous qualitative and mixed methods research on adoption,^55^ child welfare, and family rearing environments in Sweden and similar contexts.^30^ ^56^ ^57^ ^58^ ^59^ These qualitative studies provided a nuanced perspective on adoption. It was noted that national adoption in Sweden likely provided children with improved opportunities amid otherwise challenging situations, which helped guide the conceptual framing of this study.^29^ ^55^ However, it was also noted that adoption severed children from their biological history, which could increase existential distress.^60^ Although this findings was beyond the scope of our research question, it highlights the complex ethical and moral issues surrounding adoption.

## Results

### Generation 1 (biological and adoptive parents)

By design, 86.3% (n=1233) of the biological parents had at least one lifetime psychiatric disorder, 5.2% (n=74) had a suicide related record, and 42.2% (n=603) had a crime conviction or suspicion of a violent or property crime, compared with only 41.5% (n=597), 2.3% (n=33), and 5.1% (n=73) of adoptive parents, respectively (table 1). Socioeconomic disadvantage was also more common among the biological parents, with higher prevalences for receipt of social welfare (46.5% (n=664) *v* 8.8% (n=127)) and long term unemployment (19.6% (n=280) *v* 7.4% (n=106)). Comparable differences were observed in the maternal half sibling sample (table 1).

**Table 1:** Personal characteristics of parents from generation 1. Values are number (percentage)

Characteristics	Full sibling sample			Maternal half sibling sample	
	Biological parents (n=1429)	Adoptive parents (n=1438)		Biological mothers (n=2592)	Adoptive mothers (n=2680)
**Any outcome**					
No	0 (0.0)	788 (54.8)		678 (26.2)	1505 (56.2)
Yes	1429 (100.0)	650 (45.2)		1914 (73.8)	1175 (43.8)
**Any psychiatric disorder**					
No	196 (13.7)	841 (58.5)		915 (35.3)	1952 (72.8)
Yes	1233 (86.3)	597 (41.5)		1677 (64.7)	728 (27.2)
**Suicide**					
No	1355 (94.8)	1405 (97.7)		2543 (98.1)	2665 (99.4)
Yes	74 (5.2)	33 (2.3)		49 (1.9)	15 (0.6)
**Crime conviction**					
No	826 (57.8)	1365 (94.9)		2128 (82.1)	2643 (98.6)
Yes	603 (42.2)	73 (5.1)		464 (17.9)	37 (1.4)
**Highest educational level**					
Compulsory school (≤9 years)	301 (21.1)	166 (11.5)		511 (19.7)	296 (11.0)
Upper secondary school	285 (19.9)	222 (15.4)		511 (19.7)	259 (9.7)
University	54 (3.8)	148 (10.3)		80 (3.1)	154 (5.7)
Missing	789 (55.2)	902 (62.7)		1490 (57.5)	1971 (73.5)
**Ever received social welfare**					
No	765 (53.5)	1311 (91.2)		1645 (63.5)	2482 (92.6)
Yes	664 (46.5)	127 (8.8)		947 (36.5)	198 (7.4)
**Long term unemployment**					
No	1149 (80.4)	1332 (92.6)		2244 (86.6)	2570 (95.9)
Yes	280 (19.6)	106 (7.4)		348 (13.4)	110 (4.1)

Supplementary table A provides detailed definitions of parental outcomes.

### Generation 2 (adopted or remained in the biological home)

Supplementary table C displays the personal characteristics of the full sibling and maternal half sibling samples, and table 2 shows the results of the within family models. Compared with their biological full siblings who were raised by the biological parents, the adopted individuals displayed statistically significantly higher non-cognitive skills, general intelligence, and education levels, and they were statistically significantly less likely to have a psychiatric diagnosis, have a criminal conviction, or receive social welfare. Similar results were observed in the maternal half sibling sample (table 2).

**Table 2:** Within sibling cluster associations between adoption status and outcomes in the full sibling and maternal half sibling samples (generation 2). Estimates are linear beta (95% CI) unless stated otherwise

Outcomes	Full siblings (n*=*4254)			Maternal half siblings (n=7796)	
	Within sibling estimate (95% CI)	P value		Within sibling estimate (95% CI)	P value
Any psychiatric disorder	HR 0.70 (0.63 to 0.78)	<0.001*		HR 0.85 (0.78 to 0.93)	<0.001*
Criminal conviction	HR 0.66 (0.60 to 0.74)	<0.001*		HR 0.70 (0.64 to 0.76)	<0.001*
Non-cognitive skills	0.75 (0.52 to 0.99)	<0.001*		0.57 (0.37 to 0.77)	<0.001*
General intelligence	0.62 (0.40 to 0.83)	<0.001*		0.63 (0.45 to 0.81)	<0.001*
Highest education	0.52 (0.47 to 0.58)	<0.001*		0.38 (0.31 to 0.45)	<0.001*
Long term unemployment	HR 0.93 (0.85 to 1.02)	0.15		HR 0.84 (0.78 to 0.90)	<0.001*
Ever received social welfare (age >20 years)	HR 0.63 (0.57 to 0.70)	<0.001*		HR 0.65 (0.60 to 0.70)	<0.001*

CI=confidence interval; HR=hazard ratio.

* Significant after controlling for false discovery rate correction (30 tests).

### Generation 3 (offspring of generation 2)

Supplementary table D displays the personal characteristics of the offspring in the full sibling and maternal half sibling samples, and table 3 displays the results from the within cousin models. Criminal convictions and receipt of social welfare were statistically significantly less likely in the offspring of the adopted siblings. In the maternal half sibling sample, the estimates were of similar magnitude, but most associations were additionally statistically significant, likely related to being based on twice as many observations.

**Table 3:** Within cousin cluster associations between adoption status and outcomes in offspring of adopted siblings versus siblings who remained in the biological family home (generation 3). Estimates are linear beta (95% CI) unless stated otherwise

Outcome	Offspring of full siblings (n=7771)			Offspring of maternal half siblings (n=14 224)	
	Within cousin estimate (95% CI)	P value		Within cousin estimate (95% CI)	P value
Any psychiatric disorder	HR 0.94 (0.85 to 1.03)	0.18		HR 0.90 (0.83 to 0.97)	0.007*
Criminal conviction	HR 0.85 (0.76 to 0.95)	0.005*		HR 0.82 (0.74 to 0.90)	<0.001*
Non-cognitive skills	0.24 (−0.12 to 0.61)	0.19		0.30 (0.05 to 0.56)	0.02*
General intelligence	0.15 (−0.13 to 0.43)	0.28		0.29 (0.05 to 0.54)	0.02*
Highest education	0.08 (−0.01 to 0.17)	0.06		0.15 (0.07 to 0.22)	<0.001*
Upper secondary education eligibility	OR 1.01 (0.99 to 1.04)	0.34		OR 1.01 (0.99 to 1.03)	0.43
Long term unemployment	HR 0.89 (0.79 to 1.01)	0.06		HR 0.82 (0.74 to 0.91)	<0.001*
Ever received social welfare (age >20 years)	HR 0.77 (0.69 to 0.86)	<0.001*		HR 0.78 (0.71 to 0.86)	<0.001*

CI=confidence interval; HR=hazard ratio; OR=odds ratio.

* Significant after controlling for false discovery rate correction (30 tests).

### Sensitivity results

The sex stratified analyses revealed broadly similar patterns in generation 2, whereas more pronounced sex differences were observed in generation 3 (supplementary tables F and G). Specifically, in generation 3 of the full sibling sample, the associations appeared more pronounced among male siblings (and weaker among female siblings), whereas in generation 3 of the maternal half sibling sample, the associations were similar across the sexes. Furthermore, when sex was included as an additional covariate in generation 2 in both the full sibling sample and the maternal half sibling sample, the results appeared virtually identical to those of the main analyses (supplementary table E).

In stratified analyses of generation 2 by birth period (1950-65 *v* 1965-80), the associations across cohorts appeared similar to those of the main analyses (supplementary tables H and I).

Cohort stratified analyses in generation 3 showed consistency with the main analyses (supplementary tables J-L), although the associations were too imprecise to be informative among the relatively few individuals born between 1965 and 1980. Among the larger cohort born between 1980 and 1995, adoption was statistically significantly associated with lower risks of criminal convictions and receipt of social welfare, as well as higher educational attainment, particularly in the maternal half sibling sample. As fewer individuals were born from 1995 to 2010, the estimates were less precise, although they trended in the same direction as that of the main analyses.

When restricting the sample to biological mothers younger than 21 years with a firstborn child adopted, the within sibling associations remained largely consistent with the main findings across psychiatric, criminal, educational, and socioeconomic outcomes (supplementary table M). In addition, analyses examining small-for-gestational-age status provided no evidence that birth size systematically predicted adoption in either the full sibling sample or the maternal half sibling sample (supplementary table N).

We found that adoption patterns varied by birth order across family structures. Later born children were more likely to be adopted in the full sibling sample, potentially reflecting thinly stretched familial resources. In contrast, earlier born children were more likely to be adopted in the maternal half sibling sample (supplementary tables O and P and supplementary figures A and B), potentially reflecting unsuitable characteristics of partners.

When we restricted the sample to individuals relocated to their adoptive parents before age 5 years, the within sibling associations in generation 2 and within cousin associations in generation 3 were highly consistent with the main analyses in both the full sibling sample and the maternal half sibling sample (supplementary tables T and U).

Lastly, when the psychiatric diagnostic category was disaggregated into its constituent parts, the prevalence patterns were similar across the biological and adoptive parents—for example, in both groups, depression and anxiety were the most prevalent conditions, whereas psychotic disorders were rare (supplementary table Q). Furthermore, at risk families with all or none of their children adopted displayed roughly similar levels of long term unemployment and receipt of social welfare as parents with some but not all their children adopted (supplementary tables R and S).

## Discussion

Results from two samples of siblings (full siblings and maternal half siblings) agreed with those of previous quasi-experimental studies^15^ ^24^ ^25^ ^33^ ^34^ ^61^ ^62^ ^63^ and randomised controlled trials,^11^ ^12^ ^13^ showing that adopted individuals who experienced improved environmental conditions displayed better psychosocial functioning (eg, fewer criminal convictions, higher education, non-cognitive skills, and general intelligence) compared with their biological siblings who were not adopted. Furthermore, the offspring of the adopted individuals also tended to display higher psychosocial functioning, compared with their cousins (whose parents were not adopted).

### Strengths and weaknesses of this study

The main strength of this study was the adoption-discordant sibling design, which adjusted for unmeasured shared familial confounding, and the use of large population based registers covering three generations across decades of follow-up. However, residual within family confounding cannot be ruled out, including child specific characteristics, timing of adoption, or selective placement based on the characteristics of the children. Furthermore, sibling comparisons are subject to bias from carryover effects, including negative emotional consequences for biological parents who decide to have a child adopted, or that a challenging older sibling might influence the decision to have a younger, less challenging sibling adopted. However, the sensitivity analyses seemed to invalidate such interpretations, including a convergence across full and maternal half sibling samples despite different adoption patterns (later born full siblings versus earlier born maternal half siblings were more often adopted), consistent results across various subcohorts (eg, across the firstborn babies of young mothers, across those born in the 1950s versus 1970s, across sexes), and a null association with adverse birth characteristics. Additionally, historical reviews highlighted challenging external circumstances as the primary motivation for having a child adopted.^29^ ^31^ Another limitation was that within cluster analyses only applied to families in which siblings differed for adoption status, limiting generalisability to families with one child or where all or no siblings were adopted; however, at risk parents who had only one of their children adopted displayed comparable levels of unemployment and receipt of social welfare as that of at risk families who had all or none of their children adopted. In addition, the stark socioeconomic differences between biological and adoptive parents may reduce generalisability, as most real world interventions are less extensive or sustained. Finally, we lacked direct measures of child rearing processes, precluding identification of the specific mechanisms underlying the observed associations.

### Strengths and weaknesses in relation to other studies

Our findings replicate and extend previous randomised controlled trials and quasi-experiments. Unlike the Perry Preschool Project and Head Start, where the cognitive gains often diminished after the programmes,^10^ ^22^ ^23^ ^64^ adopted boys displayed higher cognitive (in addition to non-cognitive) scores at age 18 years. One explanation might be that adoption provided long term exposure to enriched home environments, in contrast with the briefer interventions in those trials. Furthermore, compared with the Perry and Abecedarian projects, our samples were substantially larger and drawn from a setting with more established safety nets, unlike that facing the more heavily disadvantaged control groups in the US trials.^7^ ^9^ ^65^ The adoptive parents were not trained, but they had higher socioeconomic status, aligning with results from interventions targeting family income,^61^ ^66^ classroom settings,^67^ ^68^ and neighbourhoods.^69^

### Meaning of the study

These findings should not be interpreted as advocating in favour of adoption as a policy intervention (which additionally requires moral, ethical, and societal considerations),^29^ ^55^ ^60^ but rather that improved rearing conditions might exert durable effects across psychiatric, educational, and socioeconomic domains, even in societies with relatively robust welfare systems. Previous work highlights multiple pathways through which early life interventions may shape later outcomes. One study emphasised that the long term effects of the Perry Preschool Project might have operated through improvements in non-cognitive skills (such as reduced externalising behaviours and enhanced academic motivation), which could influence a higher educational level and labour market performance even when previous gains on intelligence test scores had faded out.^22^ Another study suggested that improvements in physical health might constitute another important mechanism linking early childhood interventions to long term functioning.^70^ Thus, enriched family environments in childhood might operate through a combination of behavioural, motivational, health, and socioeconomic pathways.

This multi-pathway framework could also help interpret the intergenerational patterns observed in our study. Although the protective effects appeared to transfer to the third generation, the magnitude of the associations attenuated somewhat. One possibility is that, unlike their parents, the children in generation 3 were not exposed to an intervention of comparable strength. Additionally, as access to mental health services and treatment options expanded over time, the buffering role of parental socioeconomic advantage for the mental health of offspring may have diminished.^7^ ^71^

### Unanswered questions and future research

Although the results of the current study dovetailed with previous randomised controlled trials,^11^ ^12^ ^13^ ^14^ it would be beneficial to triangulate using different quasi-experimental designs that make different assumptions.^72^ Also, this study could not disentangle mechanisms, as adoptive parents outperformed biological parents across all psychosocial domains. Future research should investigate the specific mediators of benefit, such as parenting practices, income, schooling, and neighbourhood, and evaluate whether less intensive but scalable interventions could replicate these effects. Understanding how to sustain gains across generations remains a priority.

### Conclusion

Applying a quasi-experimental design to isolate unmeasured familial confounding in two large at risk sibling samples, we observed that improved home environment conditions were associated with long term gains, including some spanning several generations. These findings lend support to the lasting impact of early life interventions.

**What is already known on this topic**

Trials and quasi-experimental studies suggest that improving environments during early life can benefit later cognitive, educational, and behavioural outcomes
Evidence is, however, limited as to whether improved rearing conditions have long term effects after accounting for unmeasured familial confounding, and whether benefits extend to the next generation

**What this study adds**

In two nationwide adoption-discordant sibling samples, adopted individuals had better long term psychosocial outcomes than their unadopted biological siblings
Some of these advantages were also observed in the next generation, supporting possible intergenerational benefits of improved early rearing conditions

## Data Availability

No additional data available. The Public Access to Information and Secrecy Act in Sweden prohibits individual level data being publicly available. Researchers who are interested in replicating this study can apply for individual level data through Statistics Sweden (https://www.scb.se/en/services/ordering-data-and-statistics/ordering-microdata/) and the National Board of Health and Welfare (https://www.socialstyrelsen.se/en/statistics-and-data/registers/). The underlying code is freely available at https://github.com/ZhenxinLiao/Rearing-Conditions-and-Psychosocial-Outcomes-Across-Three-Generations-in-Sweden .

## Web extra

Extra material supplied by authors

Supplementary information: A brief history of Swedish national adoptions, adoption patterns and family structure, and additional figures A-D and tables A-Y

## Notes

Contributors: ZL and EP had full access to all the data in the study and take responsibility for the integrity of the data and the accuracy of the data analysis; they act as the guarantors. EP conceptualised the study. ZL and EP developed the methodology. ZL, MZ, IB, ZC, RKH, BMD, HL, PL, and EP contributed to the acquisition, analysis, or interpretation of the data. ZL and EP conducted the formal analysis. EP acquired the funding. RKH, MZ, and PL provided administrative, technical, or material support. EP and PL supervised the study. ZL and EP drafted the manuscript. BMD, RKH, IB, ZC, HL, PL, and EP critically reviewed and revised the manuscript. No AI tools were used in the preparation of this manuscript. The corresponding author attests that all listed authors meet authorship criteria and that no others meeting the criteria have been omitted.

Transparency: The lead authors (ZL and EP) affirm that the manuscript is an honest, accurate, and transparent account of the study being reported; that no important aspects of the study have been omitted; and that any discrepancies from the study as originally planned have been explained.

Dissemination to participants and related patient and public communities: The work will be disseminated to policymakers, professional societies, and relevant patient and public communities through multiple channels, including press releases, targeted social media outreach, presentations at stakeholder meetings, and academic conferences.

Provenance and peer review: Not commissioned; externally peer reviewed.
