# Supplementary material for: Home environment conditions during childhood and psychosocial outcomes across three generations in Sweden: population based adoption-discordant sibling comparison study
Source: BMJ. 2026 Apr 22;393:e087844. doi: 10.1136/bmj-2025-087844 (PMC13101412; doi:10.1136/bmj-2025-087844)
Supplement: Supplementary file 1 — Supplementary information: A brief history of Swedish national adoptions, adoption patterns and family structure, and additional figures A-D and tables A-Y [file liaz087844.ww.pdf]

Supplementary material

1. A brief history of Swedish national adoptions

2. Adoption patterns and family structure.

Supplementary figures

Figure A. Distribution of birth order by adoption status in full-sibling sample

Figure B. Distribution of birth order by adoption status for maternal half-sibling sample

Figure C, distribution of age at first observed co-residence for full sibling.

Figure D, distribution of age at first observed co-residence for maternal half sibling.

Supplementary tables

Table A. Description of registries and variables extracted

Table B. The ICD code, definition of outcomes, and the cut-off age for outcomes'

Table C. Demographic characteristics of adopted-away versus home-reared siblings (generation 2).

Table D. Demographic characteristics of offspring of adopted-away versus home-reared siblings (generation 3).

Table E. Within-sibling cluster associations between adoption status and outcomes in the full and maternal half-sibling samples (generation 2 controlling for birthyear & sex).

Table F. Within-sibling associations Between Adoption Status and Outcomes Among Males and females in Generation 2 and Generation 3 for full sibling sample

Table G. Within-sibling associations Between Adoption Status and Outcomes Among Males and Females in Generation 2 and Generation 3 for maternal half sibling sample

Table H. Within-sibling cluster associations between adoption status and outcomes in the full and maternal half-sibling samples (generation 2 born between 1950 and 1965).

Table I. Within-sibling cluster associations between adoption status and outcomes in the full and maternal half-sibling samples (generation 2 born between 1965 and 1980).

Table J. Within-cousin cluster associations between adoption status and outcomes in offspring of adopted-away versus home-reared siblings (generation 3 born between 1965 and 1980).

Table K. Within-cousin cluster associations between adoption status and outcomes in offspring of adopted-away versus home-reared siblings (generation 3 born between 1980 and 1995).

Table L. Within- cousin cluster associations between adoption status and outcomes in offspring of adopted-away versus home-reared siblings (generation 3 born between 1995 and 2010).

Table M. Within-sibling cluster associations between adoption status and outcomes in offspring of adopted-away versus home-reared siblings (generation 2 the firstborn child was adopted away and the biological mother was younger than 21 years at childbirth).

Table N, Association between small-for-gestational-age status and adoption in within-family models

Table O, Distribution of birth order by adoption status in full sibling sample

Table P, Distribution of birth order by adoption status in maternal half sibling sample

Table Q, Psychiatric characteristics of generations 1 of full sibling sample and maternal half-sibling sample

Table R. Demographic characteristics of Generation 1 in the full-sibling sample, comparing parents who adopted away all children versus parents who adopted away no children

Table S. Demographic characteristics of Generation 1 in the maternal half sibling sample, comparing biological mother who adopted away all children versus biological mother who adopted away no children

Table T. Within-sibling associations between adoption status and outcomes among individuals relocated before age 5 in full- and maternal half-sibling samples (Generation 2)

Table U. Within-cousin associations of adoption status with outcomes among offspring of individuals relocated before age 5 in full- and maternal half-sibling samples (Generation 3)

Table V, Incidence rates by adoption status in full siblings (generation 2)

Table W, Incidence rates by adoption status in maternal half siblings (generation 2)

Table X, Incidence rates by adoption status in full siblings (generation 3)

Table Y, Incidence rates by adoption status in maternal half siblings (generation 3)

1. A brief history of Swedish national adoptions

Sweden introduced its first adoption law in 1917, with the dual goals of (i) allowing current foster parents to become legal guardians, and (ii) improve opportunities for children living among poverty or otherwise unsuitable conditions.(1) The overarching guiding principle of this law, and the ensuing amendments, was that adoption should serve the interests of the child, although the rights of adopting parents were also considered to varying degrees up to 1970.(1) The primary motivation for adopting away a child were difficult living circumstances.(2) Most children were adopted as infants, and came to the adoption parents prior to age 1.(2) Although the courts decided on the legal matters of adoption, they relied on statements written by case officers at the Child Welfare Board. Typically, the judges followed the case workers' recommendations.(1)

The 1917 law stipulated that single individuals or married couples over the age of 25 could adopt (if they had children already, they needed special approval). The law allowed for the possibility of revoking the adoption, subject to court approval, in cases of (adoptive) parental abuse, neglect, or criminality. Pending court approval, the adoptive parents could also revoke the adoption in cases of gross misconduct toward the parents or due to the child leading a morally reprehensible or criminal life.[1]

Following a 1944 amendment, those who already had children were also allowed to adopt without special approval. Further, if the child turned out to suffer from a pre-existing physical or psychiatric condition (e.g., an intellectual disability, or "feeble-mindedness" as the stipulated at the time[1]), the adoptive parents were now allowed to revoke the adoption within 5 years.(1) Revoked adoptions were exceedingly rare, with only a handful of observed cases.(1, 2)

Up until 1958, adoptions were "weak", meaning that the adoptees inherited from their biological parents, and were only entitled to a protected share of the inheritance from their adopting parents (but not from their relatives). After 1958, adoptions become "strong", meaning that adoptees inherited from their adopting parents and all legal ties to the biological parents were severed. In the same year, it became more difficult to revoke the adoption, and from 1970 and onward, it was no longer allowed to revoke the adoption.(1)

Prior to 1953, adoptions were handled by child welfare officers, and between 1953-1973, they were handled via a public adoption agency.(2) The children were often first cared for in infant homes, which gave the biological mother an opportunity to change her mind (in the period 1950-73, 30-40% did so during this window) and for observation and medical examinations of the child.(2) Paternal consent was only required if the biological parents were married. Afterwards, the children often lived briefly with the would-be adopting parents in foster care for a short while, after which the adoption was legally approved.(2) Most adoptions occurred without the contact between the biological and adoptive parents. From the 1950s and onward, governmental

[1] Original Swedish: "sinnesslöhet"

agencies increasingly recommended that adoptive parents inform their children about their adoptive status sooner rather than later.(2)

Between 1917 and the mid-1930s, about 1,000 children were adopted each year. There was a sharp increase in adoptions during World War II, peaking at around 3,000 adoptions in 1950. Between 1950 and 1980, there was a declining trend of national adoptions, plateauing around 1,200 national adoptions per year in the 1970s.(1) Although there is no data on the matter, it is often presumed that infant adoption continued to decrease during the 1970s.(2)

The reason for the decline in adoption was threefold, all attributable to the supply (rather than demand) side. First, birth control was introduced; second, the societal safety net expanded; and, third, stigma surrounding single motherhood decreased. As a consequence, by the mid-1960s, the waiting time to adopt a child was around 5 years, and there were five willing adopting families for each available child.(1)

Several studies have examined the demographic patterns of the biological parents, the children, and the adopting parents. First, Lindgren examined court records of 54 adopted children occurring 1956-58 in Stockholm, Sweden.(1) Of these, 52 were adopted prior to age 2. Prior to formalizing the adoption, the biological mothers were given the opportunity to consult with doctors and counselors. Regarding the biological mothers, most came from lower socio-economic strata, a third had children from before, and 40% were under age 20. A similar pattern was observed among the biological fathers. Regarding the motivation to give up a child for adoption, in a review of a subsample of 19 by case worker statements from 1958, it was noted that reasons included a lack of financial resources, capabilities, or the presence of other children. Regarding the adoptive parents, all were married and most had been so for over 6 years. Almost all were childless, most came from higher socio-economic strata, and most of the mothers were housewives. Based on the case workers' descriptions, the adopting parents should not only have material wealth, but also the ability to provide a comforting, harmonious, and honest environment.(1)

Second, Bohman examined the circumstances surrounding 168 adopted children born during 2 years in the mid-1950s in Stockholm via court records and interviews.(3) Nearly all of the children were adopted prior to age 1. The majority of the biological mothers were young, unmarried, or living alone, and half had prior children. Motivations for adopting away included lack of financial resources, lack of paternal support, and stigma surrounding solo parenting. The biological fathers displayed greater rates of alcohol abuse and criminal activity, compared to representative fathers. The adoptive parents were older than typical parents, came from higher socio-economic strata, and many had struggled with infertility.(3)

Third, Vinnerljung identified 899 Swedish adopted children born 1972-1981.(4) Close to 92% were adopted prior to age 2. The biological parents had higher prevalences of psychiatric and substance use diagnoses, and less education, compared to the general population.(4)

Fourth, based on records in the City Archive in Stockholm, Nordlöf identified most adoptions registered in Stockholm 1918-1973.(2) Among these 6 737 adopted children, over 80% were legally adopted by age 4 (excluding stepchild adoptions). In a deeper review of 866 cases, Nordlöf examined the context surrounding the adoptions. Among those born 1950-1973, 99% lived with the adopting parents prior to age 5, and about 90% were legally adopted by age 4. Only 1% were adopted by a relative. Half of the biological mothers were less than 25 years old, most were from lower socio-economic strata and unmarried. Working as a maid was the most common job. For a third of the more deeply studied 866 cases, Nordlöf identified written motivations behind the adoptions. The most cited motivation was a lack of finances or housing (e.g., there were substantial housing shortages in Stockholm in the 1960s). Other reasons included ongoing education, extramarital affairs, that a new partner did not want the child in his household, or lack of resources to care for an additional child. In a few instances, the adoption was attributed to more severe conditions (e.g., rape, serious illness, intellectual disability, abandonment, or death). There were no sex differences in the adopted children. The adopting mother was often a housewife and over 80% were 30 years or older. The adopting family typically came from higher socio-economic strata.(2)

In summary, several historical reviews indicate that the vast majority of children were adopted away very early in life, primarily due to the biological mothers' lack of resources to care for the child. In contrast, the adopting parents tended to come from higher socio-economic strata.

2. Adoption patterns and family structure.

The numbers of biological and adoptive parents/mothers are not identical. This asymmetry arises from two mechanisms. First, some biological families had more than one child adopted away, and these children were sometimes placed into different adoptive families. In the full-sibling sample, among 1429 biological families, 89 families had more than one child adopted away. Specifically, 76 families had two children adopted (58 placed into different adoptive families), 10 families had three children adopted (9 placed into different adoptive families), 2 families had four children adopted into four different adoptive families, and 1 family had five children adopted into three different adoptive families.

Second, a small number of adoptive families received children originating from more than one biological family. In the full-sibling sample, among 1438 adoptive families, 19 adoptive families received children from two different biological families.

A similar pattern was observed in the maternal half-sibling sample. Among 2592 biological mothers, 172 had more than one child adopted away. Specifically, 153 families had two children adopted (126 placed into different adoptive families), 14 families had three children adopted into different adoptive families, 4 families had four children adopted into different adoptive families, and 1 family had six children adopted into six different adoptive families.

whereas among 2680 adoptive mothers, 49 received children from more than one biological family. As a result, the number of adoptive families slightly exceeds the number of biological families in the analytic samples.

We identify 406 biological mothers who appeared in both the full-sibling and maternal half-sibling samples.

We also identified 466 adoptive mothers who appeared in both the full-sibling and maternal half-sibling samples, reflecting overlapping adoption histories across family structures.

The overlap of 406 biological mothers across the full-sibling and maternal half-sibling samples reflects maternal childbearing histories spanning multiple partnership constellations. Specifically, a mother could contribute a discordant full-sibling set (i.e., children with the same biological father differing in adoption status) and, at the same time, contribute a discordant maternal half-sibling set if she had additional children with another biological father and adoption status varied across these offspring.

Figure A. Distribution of birth order by adoption status in full-sibling sample

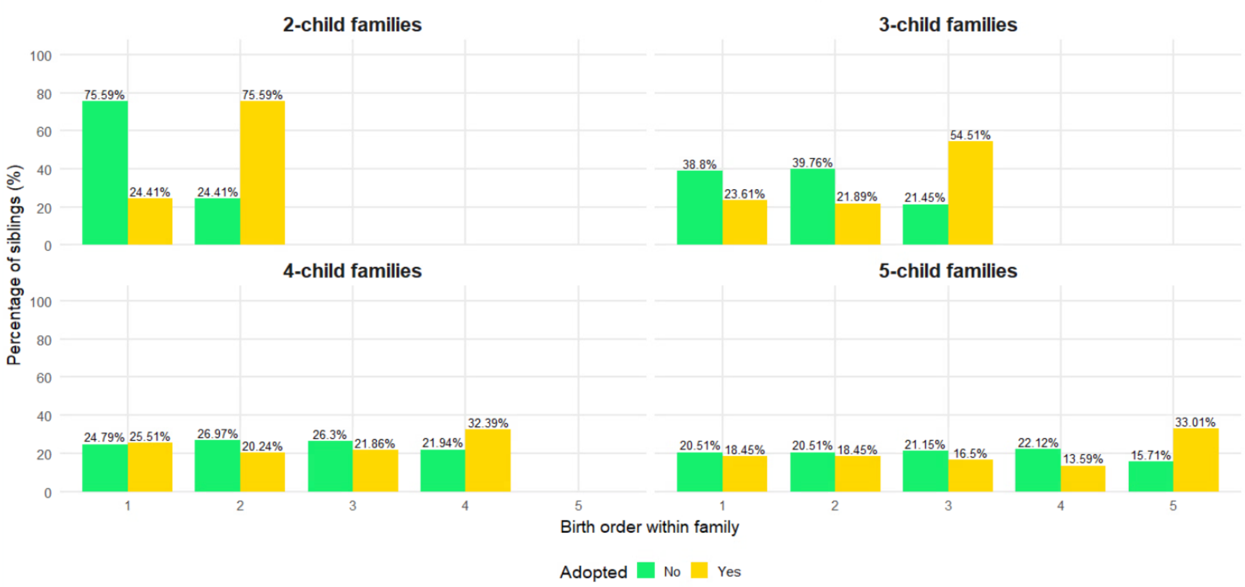

Footnote: This figure displays the percentage distribution of birth order positions among adopted (yellow) and non-adopted (green) individuals in full sibling families with two, three, and four and five children. Birth order was derived from within-family sorting by birth date.

Figure B. Distribution of birth order by adoption status for maternal half-sibling sample

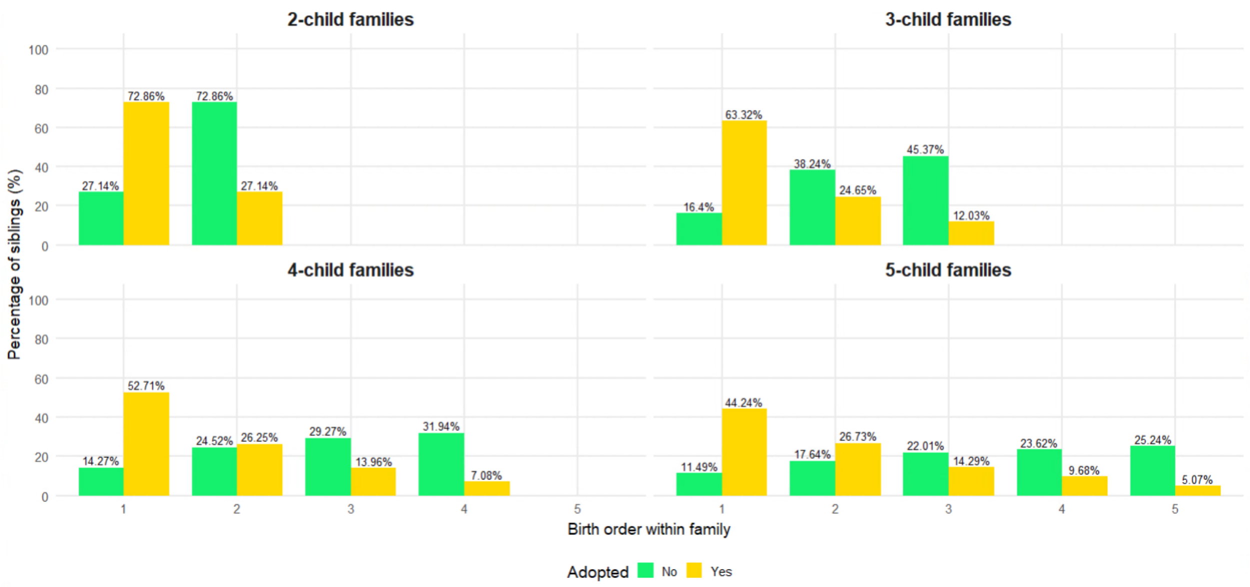

Footnote: This figure displays the percentage distribution of birth order positions among adopted (yellow) and non-adopted (green) individuals in maternal half-sibling families with two, three, four and five children. Birth order was derived from within-family sorting by birth date.

Adoption age

We constructed a proxy measure for adoption timing defined as the child's age at the first census wave in which co-residence with at least one adoptive parent was observed. Owing to the discrete timing of census data collection (i.e., it was collected every 5th year), this measure represents an approximation of adoption timing and should be interpreted as an upper bound on the true age at placement; the actual transition into the adoptive household is likely to have occurred earlier. In the full-sibling sample, the mean age at first observed co-residence with adoptive parents was 3.6 years, while in the maternal half-sibling sample the corresponding mean age was 4.1 years (supplementary figure C-D). The observed right-skewed tail beyond age five largely reflects left-truncation of the FoB data, which begin in 1960.(5) Children adopted prior to the first FoB wave, particularly those born in the 1950s, can only be observed at their age in 1960, even though adoption typically occurred earlier. Consequently, later observed ages primarily capture delayed observation rather than late placement.

Figure C, distribution of age at first observed co-residence for full sibling.

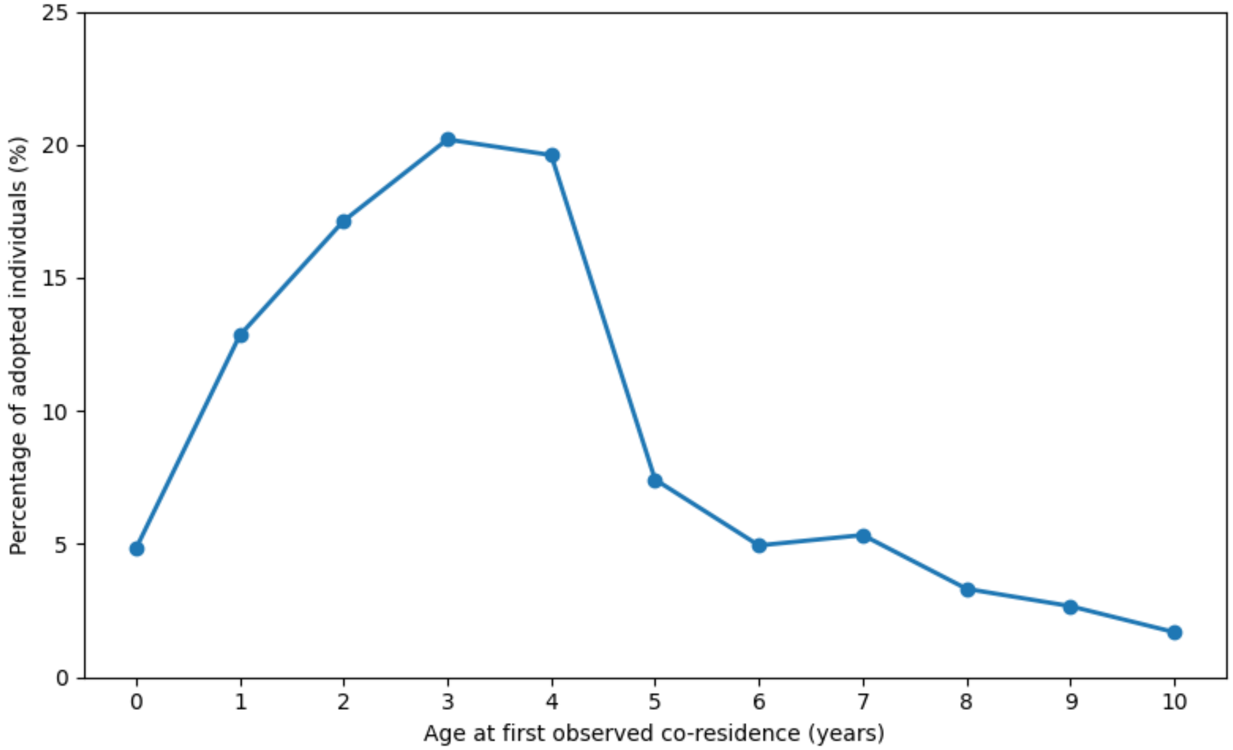

Footnote: Observations at older ages largely reflect limitations in historical census coverage, particularly for individuals born between 1950 and 1960, for whom the first available residential observation occurred at the 1960 census rather than at the time of actual relocation.

Figure D, distribution of age at first observed co-residence for maternal half sibling.

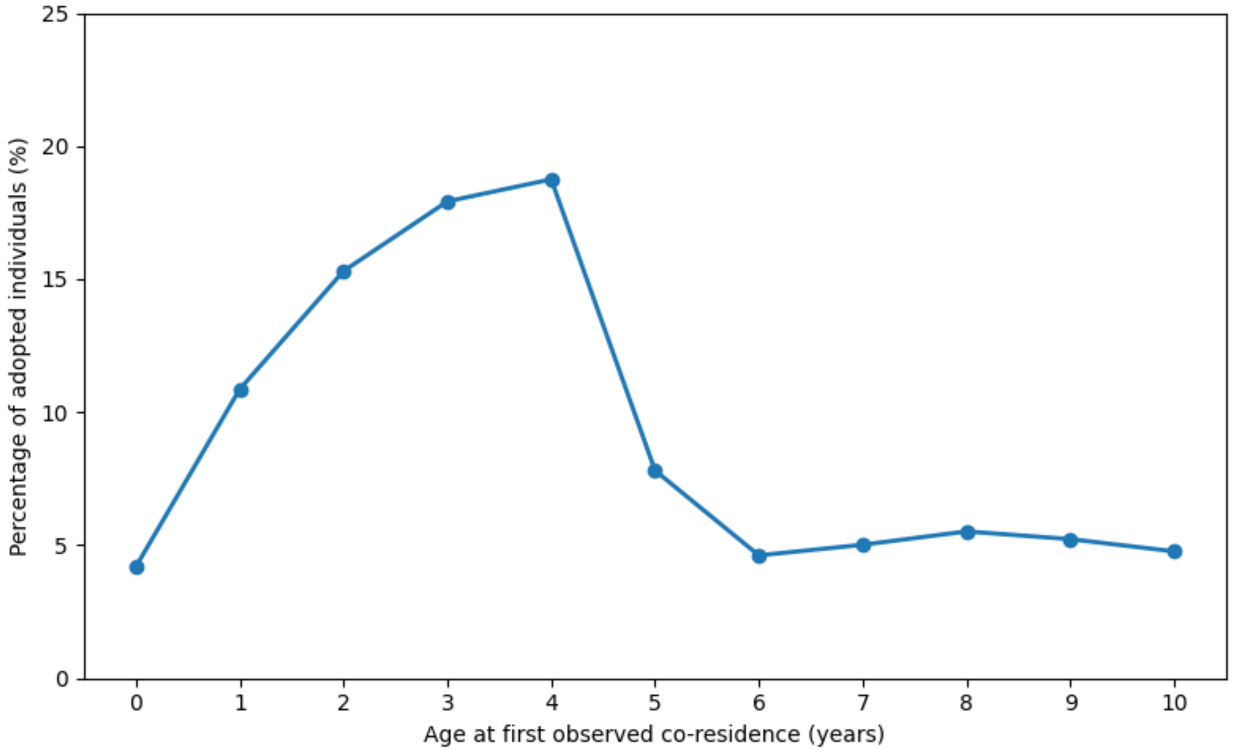

Footnote: Observations at older ages largely reflect limitations in historical census coverage, particularly for individuals born between 1950 and 1960, for whom the first available residential observation occurred at the 1960 census rather than at the time of actual relocation.

Table A. Description of registries and variables extracted

Register	Description	Variables
Total Population Register	Established in 1968 and includes demographic information (e.g., sex, age, place of birth) for the entire Swedish population. (6)	Individual identification number, Birthyear, Sex
Multi-Generation Register	Links all individuals born in Sweden since 1932 and alive in 1960 to their biological and adoptive parents. (7)	Individual identification number of individual and biological and adoptive parents
Population and Housing Censuses (FoB)	The Population and Housing Censuses (FoB) contain information on individuals, households, and dwellings. Coordinated FoB censuses were conducted in Sweden every five years between 1960 and 1990. (5)<br>In this study, FoB data were used to identify children's co-residence during the first ten years of life, allowing us to determine whether a child lived with their biological parents or with adoptive parents during early childhood.	-
National Patient Register	Includes individual-based records of psychiatric inpatient care since 1973 (complete since 1987) and psychiatric outpatient care since 2001 (complete since 2010). All diagnoses were recorded according to the International Classification of Diseases (ICD); 8th (1973/1969-1986), 9th (1987-1996), and 10th (1997-) revision. (8, 9)	Disorders, Suicide attempts
National Crime Register	Comprises all registered criminal convictions of those aged 15 and older (the age of criminal responsibility) since 1973. (10)	Court convictions of violent or property crimes
National School Register	Includes averaged junior high final grades (age 15) and (in)eligibility for high school since 1988.	Eligibility for upper secondary education
Cause of Death Register	Records all deaths in Sweden since 1952 and provides information on causes of death according to ICD. (11)	Death by suicide
Longitudinal Integration Database for Health Insurance and Market Studies	Information from the labor market and educational and social sectors for all individuals registered in Sweden over 16 years of age since 1990. (12)	Unemployment, Receipt of social welfare, Attained educational level
Swedish Military Conscription Register	The Swedish Military Conscription Register contains data on some 2 million individuals. Conscripts underwent extensive testing, data that can be used in epidemiological research. Most Swedish conscripts were born between 1951 and 1988 and tested 1969-2006. For this cohort, the register has a population coverage of about 90% for men.(13)	Non-cognitive skills, General intelligence
Swedish Twin Registry	Includes nearly all twin pairs born in Sweden from 1886 through 2000. (14)	--
National Medical Birth Register	The Medical Birth Register provides statistics on pregnancies, labour and newborns. The Register started in 1973, and it covers all pregnancies resulting in childbirth in Sweden.(15)	Gestational age, Birth weight (child, g)

Table B. The ICD code, definition of outcomes, and the cut-off age for outcomes

	ICD-8 (1969-1986)	ICD-9 (1987-1996)	ICD-10 (1997-)	Minimal Age
Any psychiatric disorder	29, 30, 31	29, 30, 31	F	-
Suicide (Suicide attempts and death by suicide)	E950-959, E980-989	E950-959, E980-989	X60-X84, Y10-Y34	10
Highest attained education	Recorded from 1990 to 2018 and treated as a continuous numeric variable, including<br>1(primary/lower secondary education, <9 years);<br>2 (primary/lower secondary education, 9 years);<br>3 (Upper secondary, <=2 years);<br>4 (Upper secondary, 3 years);<br>5 (Post secondary, <3 years);<br>6 (Post secondary, >=3 years);<br>7 (postgraduate education)			-
Eligibility for upper secondary education	Upper secondary education eligibility, 0=not eligible, 1=eligible			14
Crimes	Court convictions of violent or property crimes,<br>violent crimes: homicide (Ch 3, §1-3); assault (Ch 3, §5-6); robbery (Ch 8, §5-6); threats and violence against an officer (Ch 17, §1-2); gross violation of a person's/woman's integrity (Ch 4, §4a); unlawful coercion (Ch 4, §4); unlawful threats (Ch 4, §5); kidnapping (Ch 4, §1); illegal confinement (Ch 4, §2); arson (Ch 13, §1-2); intimidation (Ch 4, §7); sexual offence (excluding prostitution and the buying of sexual services but including child pornography) (Ch 6 §1-10, §10A, §12)<br>property crimes: theft, aggravated theft, petty theft, burglary, robbery, aggravated robbery, and attempted robbery (Ch 8, §1–4, 7–9, 12), fraud and aggravated fraud (Ch9, §6–7).			15
Non-cognitive skills	Non-cognitive skills, also called psychological functioning, this trait was assessed during the mandatory conscription evaluation following a 25-minute interview by a psychologist. The goal of the interview was to weed out those with unsuitable motives or personalities for military service, as well as measure emotional stability, persistence, social maturity, initiative, and stress tolerance.(13, 16)<br><br>The score is recorded on a nine-point stanine scale and has been widely used in Swedish register-based research as a global measure of non-cognitive ability. Previous validation studies have demonstrated good predictive validity, showing strong and independent associations with long-term educational, labor market, and health-related outcomes, even after adjustment for cognitive ability.(17) Based on 30 tape-recorded interviews, the inter-rater reliability is estimated at 0.85.(18)			18
General Intelligence scores	General Intelligence scores derived from the military conscription records (available for males only, born during 1950-1990). A battery of written tests covering verbal, spatial, logical, and technical abilities. The results were aggregated into a general cognitive ability score (G-factor).			18

	The full battery took, on average, 62 minutes to complete.(19)	
Long-term unemployment	Long-term unemployment was defined as accumulating ≥180 days of registered unemployment within a calendar year, based on annual unemployment records from the LISA register. This measure reflects annual cumulative unemployment and does not require uninterrupted or continuous unemployment spells.	18
Receipt of social welfare	Defined as having received social welfare at least once.	20

Table C. Demographic characteristics of adopted-away versus home-reared siblings (generation 2).

	Full Siblings (N=4254)		Maternal Half siblings (N=7796)	
**Characteristics**	**Home-reared**	**Adopted-away**	**Home-reared**	**Adopted-away**
**N**	2719	1535	5006	2790
**Sex**				
Male	1404 (51.6%)	757 (49.3%)	2575 (51.4%)	1464 (52.5%)
Female	1315 (48.4%)	778 (50.7%)	2431 (48.6%)	1326 (47.5%)
**Birth year**				
1950-1959	1374 (50.6%)	660 (43.0%)	1564 (31.2%)	1386 (49.7%)
1960-1969	1120 (41.2%)	719 (46.8%)	2392 (47.8%)	1188 (42.6%)
1970-1980	225 (8.3%)	156 (10.2%)	1050 (21.0%)	216 (7.7%)
**Migration**				
No	2563 (94.2%)	1434 (93.4%)	4693 (93.7%)	2631 (94.3%)
Yes	156 (5.8%)	101 (6.6%)	313 (6.3%)	159 (5.7%)
**Death**				
No	2424 (89.1%)	1397 (91.0%)	4589 (91.7%)	2535 (90.9%)
Yes	295 (10.9%)	138 (9.0%)	417 (8.3%)	255 (9.1%)
**Age at end of follow-up (Mean±SD)**	60.0 ± 6.5	59.0 ± 6.4	56.5 ± 7.2	60.1 ± 6.5
**Any psychiatric disorder**				
No	1739 (63.9%)	1077 (70.2%)	3411 (68.1%)	1964 (70.4%)
Yes	980 (36.1%)	458 (29.8%)	1595 (31.9%)	826 (29.6%)
**Crimes**				
No	1794 (66.0%)	1135 (73.9%)	3393 (67.8%)	2099 (75.2%)
Yes	925 (34.0%)	400 (26.1%)	1613 (32.2%)	691 (24.8%)
**Intelligence score (Mean±SD)**	3.8 ± 2.0	4.5 ± 2.0	4.1±2.0	4.7±2.0
**Non-cognitive skills (Mean±SD)**	3.9 ± 2.2	4.8 ± 1.9	4.2±2.0	4.7±2.0
**Highest educational level**				
Compulsory school (≤9 years)	651 (23.9%)	184 (12.0%)	938 (18.7%)	349 (12.5%)
Upper secondary school	1321 (48.6%)	784 (51.1%)	2605 (52.0%)	1389 (49.8%)
University	414 (15.2%)	399 (26.0%)	929 (18.6%)	732 (26.2%)
Missing	333 (12.3%)	168 (10.9%)	534 (10.7%)	320 (11.5%)
**Long-term unemployment**				
No	1525 (56.1%)	886 (57.7%)	2674 (53.4%)	1705 (61.1%)
Yes	1194 (43.9%)	649 (42.3%)	2332 (46.6%)	1085 (38.9%)
**Ever received social welfare (>20years old)**				
No	1400 (51.5%)	955 (62.2%)	2587 (51.7%)	1741 (62.4%)
Yes	1319 (48.5%)	580 (37.8%)	2419 (48.3%)	1049 (37.6%)

Table D. Demographic characteristics of offspring of adopted-away versus home-reared siblings (generation 3).

	Offspring of full siblings (N=7771)		Offspring of maternal half-siblings (N=14224)	
**Characteristics**	**Home-reared**	**Adopted-away**	**Home-Reared**	**Adopted-away**
**N**	5021	2750	9185	5039
**Sex**				
Male	2547 (50.7)	1406 (51.1)	4774 (52.0%)	2635 (52.3%)
Female	2474 (49.3)	1344 (48.9)	4411 (48.0%)	2404 (47.7%)
**Birth year**				
1960-1970	37 (0.7)	7 (0.3)	30 (0.3%)	16 (0.3%)
1970-1980	903 (18.0)	364 (13.2)	861 (9.4%)	799 (15.8%)
1980-1990	1850 (36.9)	984 (35.8)	2790 (30.4%)	1910 (37.9%)
1990-2000	1613 (32.1)	970 (35.3)	3210 (34.9%)	1679 (33.3%)
2000-2010	516 (10.3)	348 (12.7)	1769 (19.2%)	519 (10.3%)
2010-2020	102 (2.0)	77 (2.8)	525 (5.7%)	116 (2.3%)
**Age (Mean±SD)**	31.6 ± 9.4	30.0 ± 9.3	27.5 ± 10.0	31.1 ± 9.2
**Migration**				
No	4772 (95.0)	2586 (94.0)	8737 (95.1%)	4730 (93.9%)
Yes	249 (5.0)	164 (6.0)	448 (4.9%)	309 (6.1%)
**Death**				
No	4904 (97.7)	2704 (98.3)	9026 (98.3%)	4939 (98.0%)
Yes	117 (2.3)	46 (1.7)	159 (1.7%)	100 (2.0%)
**Any psychiatric disorder**				
No	3404 (67.8)	1936 (70.4)	6316 (68.8%)	3535 (70.2%)
Yes	1617 (32.2)	814 (29.6)	2869 (31.2%)	1504 (29.8%)
**Crimes**				
No	3865 (77.0)	2231 (81.1)	7449 (81.1%)	4115 (81.7%)
Yes	1156 (23.0)	519 (18.9)	1736 (18.9%)	924 (18.3%)
**Intelligence score (Mean±SD)**	4.4 ± 1.9	4.5 ± 1.8	4.6±1.8	4.7±1.9
**Non-cognitive skills (Mean±SD)**	4.6 ± 1.9	4.8 ± 1.8	4.5±1.8	4.6±1.8
**Highest educational level**				
Compulsory school (≤9 years)	817 (16.3)	435 (15.8)	1666 (18.1%)	699 (13.9%)
Upper secondary school	2407 (47.9)	1228 (44.7)	3829 (41.7%)	2220 (44.1%)
University	1237 (24.6)	729 (26.5)	2024 (22.0%)	1557 (30.9%)
Missing *	560 (11.2)	358 (13.0)	1666 (18.1%)	563 (11.2%)
**Upper secondary education eligibility**				
No	618 (12.3)	337 (12.3)	1101 (12.0%)	530 (10.5%)
Yes	2719 (54.1)	1625 (59.1)	5396 (58.7%)	2984 (59.2%)
Missing *	1684 (33.6)	788 (28.7)	2688 (29.3%)	1525 (30.3%)
**Long-term unemployment**				
No	3995 (79.6)	2298 (83.6)	7805 (85.0%)	4172 (82.8%)
Yes	1026 (20.4)	452 (16.4)	1380 (15.0%)	867 (17.2%)
**Ever received social welfare (>20years old)**				

No	3650 (72.7)	2177 (79.2)	7369 (80.2%)	4010 (79.6%)
Yes	1371 (27.3)	573 (20.8)	1816 (19.8%)	1029 (20.4%)

* Educational attainment was derived from the Longitudinal Integration Database for Health Insurance and Labour Market Studies (LISA), available for individuals aged ≥16 years in Sweden from 1990 onwards; therefore, this measure is unavailable for earlier birth cohorts.

Estimates for upper secondary education eligibility was obtained from the Swedish National School Register, which includes information on junior high school grades and eligibility for upper secondary education from 1988 onward. Consequently, this measure is not available for earlier birth cohorts.

Table E. Within-sibling cluster associations between adoption status and outcomes in the full and maternal half-sibling samples (generation 2 controlling for birthyear & sex).

**Outcomes**	**Full siblings (*N*=4254)**		**Maternal half-siblings (*N*=7796)**	
	**Within-sibling HR/linear beta (95%CI)**	**p-value**	**Within-sibling HR/ linear beta (95%CI)**	**p-value**
**Any psychiatric disorder** (HR)	0.70 (0.63, 0.78)	<0.001*	0.85 (0.78, 0.93)	<0.001*
**Crimes** (HR)	0.65 (0.58, 0.73)	<0.001*	0.66 (0.60, 0.72)	<0.001*
**Non-cognitive skills** (linear beta)	0.76 (0.52, 0.99)	<0.001*	0.58 (0.38, 0.78)	<0.001*
**General intelligence** (linear beta)	0.61 (0.40, 0.82)	<0.001*	0.63 (0.45, 0.82)	<0.001*
**Highest education** (linear beta)	0.52 (0.43, 0.60)	<0.001*	0.39 (0.32, 0.46)	<0.001*
**Long-term unemployment** (HR)	0.94 (0.85, 1.03)	0.169	0.83 (0.77, 0.89)	<0.001*
**Ever received social welfare** (>20years old; HR)	0.63 (0.57, 0.70)	<0.001*	0.65 (0.61, 0.70)	<0.001*

* indicates statistical significance at $p < 0.05$.

Table F. Within-sibling associations Between Adoption Status and Outcomes Among Males and females in Generation 2 and Generation 3 for full sibling sample

Full sibling sample outcomes N(%)	Generation 2: males (N=2161)		Generation 3: males (N=3953)		Generation 2: females (N=2093)		Generation 3: females (N=3818)	
	HR//OR/linear beta(95%CI)	p	HR/OR/linear beta (95%CI)	p	HR/OR/linear beta(95%CI)	p	HR/OR/linear beta (95%CI)	p
**Any psychiatric disorder** (HR)	0.74 (0.61, 0.88)	0.001*	0.92 (0.80, 1.06)	0.249	0.69 (0.58, 0.83)	<0.001*	1.04 (0.90, 1.20)	0.630
**Crimes** (HR)	0.63 (0.54, 0.75)	<0.001*	0.79 (0.68, 0.92)	0.002*	0.81 (0.64, 1.02)	0.077	0.93 (0.75, 1.15)	0.480
**Non-cognitive skills** (linear beta)	0.76 (0.52, 0.99)	<0.001*	0.25 (−0.12, 0.63)	0.186	-	-	-	-
**General intelligence** (linear beta)	0.61 (0.40, 0.82)	<0.001*	0.12 (−0.17, 0.41)	0.421	-	-	-	-
**Highest education** (linear beta)	0.42 (0.27, 0.58)	<0.001*	0.12 (−0.17, 0.41)	0.421	0.56 (0.42, 0.71)	<0.001*	0.07 (−0.07, 0.20)	0.330
**Upper secondary education eligibility** (OR)	-	-	0.99 (0.95, 1.04)	0.604	-	-	1.02 (0.98, 1.07)	0.270
**Long-term unemployment** (HR)	1.20 (1.03, 1.40)	0.017*	0.87 (0.73, 1.03)	0.100	0.75 (0.63, 0.90)	0.002*	1.03 (0.85, 1.24)	0.760
**Ever received social welfare** (>20years old; HR)	0.65 (0.55, 0.78)	<0.001*	0.75 (0.64, 0.88)	<0.001*	0.65 (0.55, 0.77)	<0.001*	0.93 (0.79, 1.09)	0.390

* indicates statistical significance at $p < 0.05$.

[a]Estimates for non-cognitive skills and general intelligence are available for males only. These outcomes are based on data from the Swedish Military Conscription Register, which administered standardized psychological and cognitive assessments to approximately 90% of men in the relevant birth cohorts. No comparable assessments were conducted for women; therefore, female estimates are not reported.

[b] Estimates for upper secondary education eligibility are not available for the 1950–1980 birth cohort (generation 2) because this outcome was obtained from the Swedish National School Register, which includes information on junior high school grades and eligibility for upper secondary education from 1988 onward. Consequently, this measure is not available for earlier birth cohorts.

Table G. Within-sibling associations Between Adoption Status and Outcomes Among Males and Females in Generation 2 and Generation 3 for maternal half sibling sample

Maternal half sibling outcomes N(%)	Generation 2: males (N=4039)		Generation 3: males (N=7409)		Generation 2: females (N=3757)		Generation 3: females (N=6815)	
	HR/OR/linear beta(95%CI)	p	HR/OR/ linear beta (95%CI)	p	HR/OR/linear beta(95% CI)	p	HR/OR/linear beta (95%CI)	p
**Any psychiatric disorder** (HR)	0.87 (0.75, 1.00)	0.050	0.89 (0.79, 1.00)	0.043*	0.98 (0.84, 1.13)	0.753	0.93 (0.83, 1.05)	0.249
**Crimes** (HR)	0.62 (0.54, 0.70)	<0.001*	0.80 (0.70, 0.90)	<0.001*	0.72 (0.59, 0.88)	<0.001*	0.80 (0.67, 0.96)	0.014*
**Non-cognitive skills** (linear beta)	0.58 (0.37, 0.78)	<0.001*	0.32 (0.05, 0.58)	0.020*	-	-	-	-
**General intelligence** (linear beta)	0.63 (0.44, 0.82)	<0.001*	0.30 (0.05, 0.55)	0.017*	-	-	-	-
**Highest education** (linear beta)	0.26 (0.13, 0.38)	<0.001*	0.12 (0.02, 0.22)	0.017*	0.54 (0.42, 0.66)	<0.001*	0.13 (0.02, 0.24)	0.019*
**Upper secondary education eligibility** (OR)	-	-	1.01 (0.97, 1.05)	0.635	-	-	1.00 (0.96, 1.03)	0.780
**Long-term unemployment** (HR)	0.89 (0.78, 1.00)	0.058	0.82 (0.71, 0.95)	0.007*	0.85 (0.75, 0.98)	0.021*	0.77 (0.64, 0.93)	0.006*
**Ever received social welfare** (>20years old; HR)	0.64 (0.57, 0.73)	<0.001*	0.81 (0.70, 0.92)	0.002*	0.75 (0.66, 0.85)	<0.001*	0.80 (0.69, 0.93)	0.003*

* indicates statistical significance at $p < 0.05$.

[a]Estimates for non-cognitive skills and general intelligence are available for males only. These outcomes are based on data from the Swedish Military Conscription Register, which administered standardized psychological and cognitive assessments to approximately 90% of men in the relevant birth cohorts. No comparable assessments were conducted for women; therefore, female estimates are not reported.

[b] Estimates for upper secondary education eligibility are not available for the 1950–1980 birth cohort (generation 2) because this outcome was obtained from the Swedish National School Register, which includes information on junior high school grades and eligibility for upper secondary education from 1988 onward. Consequently, this measure is not available for earlier birth cohorts.

Table H. Within-sibling cluster associations between adoption status and outcomes in the full and maternal half-sibling samples (generation 2 born between 1950 and 1965).

Outcomes	Full siblings (*N*=3199)		Maternal half-siblings (*N*=5331)	
	Within-sibling HR/linear beta (95%CI)	p-value	Within-sibling HR/ linear beta (95%CI)	p-value
**Any psychiatric disorder** (HR)	0.69 (0.61, 0.79)	<0.001*	0.84 (0.76, 0.94)	0.001*
**Crimes** (HR)	0.64 (0.56, 0.74)	<0.001*	0.73 (0.65, 0.81)	<0.001*
**Non-cognitive skills** (linear beta)	0.72 (0.41, 1.02)	<0.001*	0.51 (0.23, 0.78)	<0.001*
**General intelligence** (linear beta)	0.64 (0.37, 0.91)	<0.001*	0.62 (0.36, 0.87)	<0.001*
**Highest education** (linear beta)	0.52 (0.41, 0.63)	<0.001*	0.42 (0.33, 0.52)	<0.001*
**Long-term unemployment** (HR)	0.94 (0.84, 1.06)	0.340	0.83 (0.76, 0.91)	<0.001*
**Ever received social welfare** (>20years old; HR)	0.72 (0.64, 0.81)	<0.001*	0.69 (0.63, 0.76)	<0.001*

* indicates statistical significance at $p < 0.05$.

Table I. Within-sibling cluster associations between adoption status and outcomes in the full and maternal half-sibling samples (generation 2 born between 1965 and 1980).

Outcomes	Full siblings (*N*=1055)		Maternal half-siblings (*N*=2465)	
	Within-sibling HR/linear beta (95%CI)	p-value	Within-sibling HR/ linear beta (95%CI)	p-value
**Any psychiatric disorder** (HR)	0.71 (0.55, 0.90)	0.005*	0.99 (0.80, 1.24)	0.980
**Crimes** (HR)	0.75 (0.58, 0.95)	0.019*	0.53 (0.43, 0.66)	<0.001*
**Non-cognitive skills** (linear beta)	0.88 (0.35, 1.40)	0.001*	1.00 (0.56, 1.45)	<0.001*
**General intelligence** (linear beta)	0.84 (0.36, 1.32)	<0.001*	0.74 (0.33, 1.15)	<0.001*
**Highest education** (linear beta)	0.53 (0.31, 0.75)	<0.001*	0.25 (0.07, 0.43)	0.007*
**Long-term unemployment** (HR)	0.91 (0.75, 1.11)	0.350	0.73 (0.61, 0.86)	<0.001*
**Ever received social welfare** (>20years old; HR)	0.48 (0.38, 0.60)	<0.001*	0.48 (0.40, 0.57)	<0.001*

* indicates statistical significance at $p < 0.05$.

Table J. Within-cousin cluster associations between adoption status and outcomes in offspring of adopted-away versus home-reared siblings (generation 3 born between 1965 and 1980).

Outcome	Offspring of full siblings (N=1311)		Offspring of maternal half-siblings (N=1706)	
	Within-cousin HR/OR/linear beta(95%CI)	p-value	Within-cousin HR/OR/linear beta (95%CI)	p-value
**Any psychiatric disorder** (HR)	1.02 (0.70, 1.48)	0.924	0.94 (0.70, 1.27)	0.710
**Crimes** (HR)	1.23 (0.84, 1.80)	0.294	0.96 (0.71, 1.30)	0.810
**Non-cognitive skills** (linear beta)	−0.40 (−1.23, 0.42)	0.338	0.30 (-0.25, 0.86)	0.283
**General intelligence** (linear beta)	−0.12 (−0.75, 0.51)	0.711	0.24 (-0.26, 0.73)	0.346
**Highest education** (linear beta)	0.20 (−0.08, 0.48)	0.161	0.08 (-0.17, 0.33)	0.528
**Upper secondary education eligibility** (OR)*	-	-	-	-
**Long-term unemployment** (HR)	1.10 (0.82, 1.46)	0.526	0.81 (0.64, 1.02)	0.074
**Ever received social welfare** (>20years old; HR)	1.04 (0.79, 1.37)	0.774	0.69 (0.54, 0.87)	0.002*

* indicates statistical significance at $p < 0.05$. Estimates for upper secondary education eligibility are not available for the 1965–1980 birth cohort because this outcome was obtained from the Swedish National School Register, which includes information on junior high school grades and eligibility for upper secondary education from 1988 onward. Consequently, this measure is not available for earlier birth cohorts.

Table K. Within-cousin cluster associations between adoption status and outcomes in offspring of adopted-away versus home-reared siblings (generation 3 born between 1980 and 1995).

Outcome	Offspring of full siblings (N=4473)		Offspring of maternal half-siblings (N=7733)	
	Within-cousin HR/OR/linear beta(95%CI)	p-value	Within-cousin HR/OR/linear beta (95%CI)	p-value
**Any psychiatric disorder** (HR)	0.98 (0.86, 1.12)	0.779	0.88 (0.79, 0.98)	0.019*
**Crimes** (HR)	0.86 (0.74, 1.00)	0.045*	0.74 (0.65, 0.84)	<0.001*
**Non-cognitive skills** (linear beta)	0.75 (0.23, 1.27)	0.005*	0.30 (-0.08, 0.68)	0.118
**General intelligence** (linear beta)	0.12 (−0.29, 0.53)	0.573	0.38 (0.03, 0.73)	0.033*
**Highest education** (linear beta)	0.12 (0.00, 0.24)	0.048*	0.19 (0.10, 0.29)	<0.001*
**Upper secondary education eligibility** (OR)	1.01 (0.97, 1.04)	0.673	1.03 (1.00, 1.06)	0.075
**Long-term unemployment** (HR)	0.89 (0.75, 1.05)	0.156	0.88 (0.77, 1.02)	0.084
**Ever received social welfare** (>20years old; HR)	0.82 (0.71, 0.94)	0.006*	0.86 (0.76, 0.97)	0.018*

* indicates statistical significance at $p < 0.05$.

Table L. Within-cousin cluster associations between adoption status and outcomes in offspring of adopted-away versus home-reared siblings (generation 3 born between 1995 and 2010).

Outcome	Offspring of full siblings (N=1808)		Offspring of maternal half-siblings (N=4144)	

	Within-cousin HR/OR/linear beta(95%CI)	p-value	Within-cousin HR/OR/linear beta (95%CI)	p-value
**Any psychiatric disorder** (HR)	0.79 (0.62, 1.00)	0.051	0.83 (0.69, 1.00)	0.055
**Crimes** (HR)	0.66 (0.43, 1.02)	0.062	1.13 (0.81, 1.59)	0.458
**Non-cognitive skills** (linear beta)*	-	-	-	-
**General intelligence** (linear beta)*	-	-	-	-
**Highest education** (linear beta)	−0.04 (−0.26, 0.17)	0.708	-0.04 (-0.21, 0.13)	0.681
**Upper secondary education eligibility** (OR)	1.20 (1.00, 1.44)	0.045*	0.97 (0.92, 1.03)	0.299
**Long-term unemployment** (HR)	0.87 (0.30, 2.55)	0.800	1.54 (0.72, 3.31)	0.267
**Ever received social welfare** (>20years old; HR)	0.49 (0.27, 0.90)	0.022*	0.66 (0.37, 1.21)	0.181

* indicates statistical significance at $p < 0.05$. Estimates for non-cognitive skills and general intelligence are not available for the 1995–2010 birth cohort because these measures were obtained from the Swedish Military Conscription Register, which includes individuals born primarily between 1951 and 1988 and tested between 1969 and 2006. Consequently, no data are available for later birth cohorts.

Table M. Within-sibling cluster associations between adoption status and outcomes in offspring of adopted-away versus home-reared siblings (generation 2 the firstborn child was adopted away and the biological mother was younger than 21 years at childbirth).

Outcomes	Full siblings (*N*=1104)		Maternal half-siblings (*N*=2520)	
	Within-sibling HR/linear beta (95%CI)	p-value	Within-sibling HR/ linear beta (95%CI)	p-value
**Any psychiatric disorder** (HR)	0.74 (0.57, 0.95)	0.018*	0.95 (0.77, 1.17)	0.636
**Crimes** (HR)	0.37 (0.27, 0.51)	<0.001*	0.76 (0.61, 0.94)	0.011*
**Non-cognitive skills** (linear beta)	0.63 (0.04, 1.22)	0.035*	-0.06 (-0.58, 0.46)	0.828
**General intelligence** (linear beta)	0.86 (0.22, 1.51)	0.009*	0.69 (0.25, 1.12)	0.002*
**Highest education** (linear beta)	0.37 (0.15, 0.58)	0.001*	0.26 (0.10, 0.43)	0.002*
**Long-term unemployment** (HR)	0.95 (0.76, 1.20)	0.694	0.76 (0.64, 0.91)	0.003*
**Ever received social welfare** (>20years old; HR)	0.69 (0.55, 0.87)	0.002*	0.59 (0.49, 0.70)	<0.001*

* indicates statistical significance at $p < 0.05$.

To examine whether adoption decisions were systematically related to observable birth endowments, we conducted a sensitivity analysis focusing on small for gestational age (SGA). SGA was defined as birth weight below the sex-specific 10th percentile within strata of gestational age, grouped by completed weeks, using information from the Swedish Medical Birth Register. Gestational age was measured in completed days based on the register's best estimate, and births were restricted to individuals born from 1973 onwards, when detailed perinatal information became available.

We assessed whether being born SGA predicted adoption by estimating within-family logistic regression models in which adoption status was regressed on the SGA indicator, with family fixed effects and standard errors clustered at the family level. In the full-sibling sample (77 informative individuals from 34 families with within-family variation in SGA), being born SGA was not significantly associated with adoption (log-odds coefficient = 0.42, SE = 1.06; p = 0.69). In the maternal half-sibling sample (122 individuals from 54 families), estimates were close to the null and similarly imprecise (log-odds coefficient = 1.47, SE = 1.11; p = 0.18). Overall, we have limited statistical power, and the results provide no evidence that adoption decisions were systematically driven by adverse observable birth endowments.

Table N, Association between small-for-gestational-age status and adoption in within-family models

Sample	Informative individuals (families)	Log-odds coefficient (SGA → adoption)	Standard error	p-value
Full siblings	77 (34 families)	0.42	1.06	0.69
Maternal half-siblings	122 (54 families)	1.47	1.11	0.18

*Small for gestational age (SGA) was defined as birth weight below the sex-specific 10th percentile within strata of gestational age (grouped by completed weeks), based on data from the Swedish Medical Birth Register. Analyses were restricted to individuals born from 1973 onwards, when detailed perinatal information became available. Estimates were obtained from within-family logistic regression models with family fixed effects and standard errors clustered at the family level. Only families with within-family variation in SGA contributed to the estimation. Due to the limited number of informative families, statistical power was low.

Table O, Distribution of birth order by adoption status in full sibling sample

Number of children in family	Adoption	Birth order 1	Birth order 2	Birth order 3	Birth order 4	Birth order 5
2	No	511 (75.59%)	165 (24.41%)	—	—	—
2	Yes	165 (24.41%)	511 (75.59%)	—	—	—
3	No	322 (38.80%)	330 (39.76%)	178 (21.45%)	—	—
3	Yes	110 (23.61%)	102 (21.89%)	254 (54.51%)	—	—
4	No	148 (24.79%)	161 (26.97%)	157 (26.30%)	131 (21.94%)	—
4	Yes	63 (25.51%)	50 (20.24%)	54 (21.86%)	80 (32.39%)	—
5	No	64 (20.51%)	64 (20.51%)	66 (21.15%)	69 (22.12%)	49 (15.71%)
5	Yes	19 (18.45%)	19 (18.45%)	17 (16.50%)	14 (13.59%)	34 (33.01%)

Table P, Distribution of birth order by adoption status in maternal half sibling sample

Number of children in family	Adoption	Birth order 1	Birth order 2	Birth order 3	Birth order 4	Birth order 5
2	No	288 (27.14%)	773 (72.86%)	—	—	—
2	Yes	773 (72.86%)	288 (27.14%)	—	—	—
3	No	292 (16.40%)	681 (38.24%)	808 (45.37%)	—	—
3	Yes	637 (63.32%)	248 (24.65%)	121 (12.03%)	—	—
4	No	177 (14.27%)	304 (24.52%)	363 (29.27%)	396 (31.94%)	—
4	Yes	253 (52.71%)	126 (26.25%)	67 (13.96%)	34 (7.08%)	—
5	No	71 (11.49%)	109 (17.64%)	136 (22.01%)	146 (23.62%)	156 (25.24%)
5	Yes	96 (44.24%)	58 (26.73%)	31 (14.29%)	21 (9.68%)	11 (5.07%)

Table Q, Psychiatric characteristics of generations 1 of full sibling sample and maternal half-sibling sample

	Full-sibling sample		Maternal half-sibling sample	
**Parental characteristics N(%)**	**Biological parents**	**Adoptive parents**	**Biological mothers**	**Adoptive mothers**
Depression	25.8	12.1	22.8	7.5
Anxiety	16.7	7.0	16.4	4.6
OCD*	0.3	0.1	0.3	<0.1
PTSD*	6.1	1.3	5.0	1.1
Bipolar disorder	3.2	1.9	2.7	1.1
Schizophrenia	3.1	0.3	1.7	0.3
Alcohol use disorder	37.4	4.4	13.3	1.2
Drug use disorder	14.2	2.8	11.5	1.6

*OCD: Obsessive–Compulsive Disorder; PTSD: post-traumatic stress disorder.

Table R. Demographic characteristics of Generation 1 in the full-sibling sample, comparing parents who adopted away all children versus parents who adopted away no children

Parental characteristics	Parents adopt away all kids (N = 269)	Parents adopt away no kids (N = 556762)
**Any outcome[c]**		
No	0 (0.0)	0 (0.0)
Yes	269 (100.0)	556,762 (100.0)
**Any psychiatric disorder**		
No	31 (11.5)	66,665 (12.0)
Yes	238 (88.5)	490,097 (88.0)
**Suicide**		
No	249 (92.6)	534,575 (96.0)
Yes	20 (7.4)	22,187 (4.0)
**Crime**		
No	144 (53.5)	424,160 (76.2)
Yes	125 (46.5)	132,602 (23.8)
**Highest educational level**		
Compulsory school (≤9 years)	54 (20.1)	91,526 (16.4)
Upper secondary school	36 (13.4)	155,870 (28.0)
University	21 (7.8)	93,364 (16.8)
Missing	166 (61.7)	216,002 (38.8)
**Ever received social welfare**		
No	149 (55.4)	432,443 (77.7)
Yes	120 (44.6)	124,319 (22.3)
**Long-term unemployment[b]**		
No	209 (77.7)	428,923 (77.0)
Yes	60 (22.3)	127,839 (23.0)

N[a] in the full sibling sample is the number of pairs for biological parents and adoptive parents. b Long-term unemployment was defined as an unemployment status of 180 days or more. c Included any psychiatric disorders, suicide, and crime.

Table S. Demographic characteristics of Generation 1 in the maternal half sibling sample, comparing biological mother who adopted away all children versus biological mother who adopted away no children

Parental characteristics	Mothers adopt away all kids (N = 38)	Mothers adopt away no kids (N =55419)
**Any outcome[c]**		
No	6 (15.8)	20,908 (37.7)
Yes	32 (84.2)	34,511 (62.3)
**Any psychiatric disorder**		
No	10 (26.3)	25,042 (45.2)
Yes	28 (73.7)	30,377 (54.8)
**Suicide**		
No	36 (94.7)	54,685 (98.7)
Yes	2 (5.3)	734 (1.3)
**Crime**		
No	31 (81.6)	47,881 (86.4)
Yes	7 (18.4)	7,538 (13.6)
**Highest educational level**		
Compulsory school (≤9 years)	3 (7.9)	11,736 (21.2)
Upper secondary school	5 (13.2)	16,753 (30.2)
University	1 (2.6)	5,119 (9.2)
Missing	29 (76.3)	21,811 (39.4)
**Ever received social welfare**		
No	26 (68.4)	32,907 (59.4)
Yes	12 (31.6)	22,512 (40.6)
**Long-term unemployment[b]**		
No	36 (94.7)	44,171 (79.7)
Yes	2 (5.3)	11,248 (20.3)

N[a] in the full sibling sample is the number of pairs for biological parents and adoptive parents. b Long-term unemployment was defined as an unemployment status of 180 days or more. c Included any psychiatric disorders, suicide, and crime. For maternal half-siblings, any outcomes for biological mothers do not sum to 100% because the definition of a high-risk rearing environment incorporates information from both parents. In cases where risk is identified via the father, the biological mother may not meet the criterion individually. When considering parents jointly (i.e., either parent meeting the criterion), the presence of any outcome is 100%.

Table T. Within-sibling associations between adoption status and outcomes among individuals relocated before age 5 in full- and maternal half-sibling samples (Generation 2)

Outcomes	Full siblings (*N*=3514)		Maternal half-siblings (*N*=5807)	
	Within-sibling HR/linear beta (95%CI)	p-value	Within-sibling HR/ linear beta (95%CI)	p-value
**Any psychiatric disorder** (HR)	0.66 (0.59, 0.75)	<0.001*	0.83 (0.76, 0.92)	<0.001*
**Crimes** (HR)	0.65 (0.58, 0.74)	<0.001*	0.68 (0.62, 0.75)	<0.001*
**Non-cognitive skills** (linear beta)	0.81 (0.55, 1.07)	<0.001*	0.74 (0.53, 0.96)	<0.001*
**General intelligence** (linear beta)	0.59 (0.35, 0.83)	<0.001*	0.69 (0.49, 0.89)	<0.001*
**Highest education** (linear beta)	0.53 (0.44, 0.63)	<0.001*	0.37 (0.29, 0.45)	<0.001*
**Long-term unemployment** (HR)	0.91 (0.82, 1.00)	0.053	0.81 (0.75, 0.88)	<0.001*
**Ever received social welfare** (>20years old; HR)	0.59 (0.53, 0.66)	<0.001*	0.59 (0.55, 0.65)	<0.001*

* indicates statistical significance at $p < 0.05$.

Table U. Within-cousin associations of adoption status with outcomes among offspring of individuals relocated before age 5 in full- and maternal half-sibling samples (Generation 3)

Outcome	Offspring of full siblings (*N*=6339)		Offspring of maternal half-siblings (*N*=9944)	
	Within-cousin HR/OR/linear beta(95%CI)	p-value	Within-cousin HR/OR/linear beta (95%CI)	p-value
**Any psychiatric disorder** (HR)	0.91 (0.82, 1.02)	0.093	0.88 (0.80, 0.96)	0.005*
**Crimes** (HR)	0.81 (0.71, 0.92)	0.002*	0.80 (0.72, 0.90)	<0.001*
**Non-cognitive skills** (linear beta)	0.24 (-0.26, 0.74)	0.352	0.33 (-0.03, 0.69)	0.07
**General intelligence** (linear beta)	0.16 (-0.22, 0.54)	0.415	0.52 (0.20, 0.85)	0.002*
**Highest education** (linear beta)	0.12 (0.02, 0.21)	0.021*	0.20 (0.12, 0.28)	<0.001*
**Upper secondary education eligibility** (OR)*	1.01 (0.98, 1.04)	0.357	1.01 (0.99, 1.04)	0.353
**Long-term unemployment** (HR)	0.83 (0.71, 0.98)	0.026*	0.76 (0.66, 0.87)	<0.001*
**Ever received social welfare** (>20years old; HR)	0.70 (0.62, 0.80)	<0.001*	0.76 (0.67, 0.85)	<0.001*

* indicates statistical significance at $p < 0.05$.

Table V, Incidence rates by adoption status in full siblings (generation 2)

Outcome	Adoption	Events	Person-years (PY)	Incidence rate (per 10,000 PY)
**Any psychiatric disorder**	No	952	138,965.20	68.5
	Yes	442	79,502.40	56
**Crimes**	No	904	128,044.80	70.6
	Yes	388	76,624.50	50.6
**Ever received social welfare >20y**	No	1290	126,052.10	102.3
	Yes	564	74,703.70	75.5
**Short-term unemployment**	No	1165	132,241.30	88.1
	Yes	634	74,011.00	85.7

Table W, Incidence rates by adoption status in maternal half siblings (generation 2)

Outcome	Adoption	Events	Person-years (PY)	Incidence rate (per 10,000 PY)
**Any psychiatric disorder**	No	1552	245,066.60	63.3
	Yes	807	147,612.70	54.7
**Crimes**	No	1568	226,981.70	69.1
	Yes	674	142,600.70	47.3
**Ever received social welfare >20y**	No	2354	216,846.50	108.6
	Yes	1021	138,950.80	73.5
**Short-term unemployment**	No	2274	223,918.60	101.6
	Yes	1065	140,431.00	75.8

Table X, Incidence rates by adoption status in full siblings (generation 3)

Outcome	Adoption	Events	Person-years (PY)	Incidence rate (per 10,000 PY)

**Any psychiatric disorder**	No	1584	139,061.60	113.9
	Yes	795	72,439.00	109.7
**Crimes**	No	1119	139,187.70	80.4
	Yes	504	73,689.90	68.4
**Ever received social welfare >20y**	No	1335	138,125.60	96.7
	Yes	556	73,882.60	75.3
**Short-term unemployment**	No	999	143,032.50	69.8
	Yes	442	75,807.20	58.3

Table Y, Incidence rates by adoption status in maternal half siblings (generation 3)

Outcome	Adoption	Events	Person-years (PY)	Incidence rate (per 10,000 PY)
**Any psychiatric disorder**	No	2822	221,050.70	127.7
	Yes	1468	137,449.90	106.8
**Crimes**	No	1662	226,475.60	73.4
	Yes	884	140,424.60	63
**Ever received social welfare >20y**	No	1778	227,603.90	78.1
	Yes	997	139,717.30	71.4
**Short-term unemployment**	No	1331	234,364.10	56.8
	Yes	842	143,002.00	58.9

References:

1. Lindgren C. En riktig familj: Adoption, föräldraskap och barnets bästa 1917-1975. Stockholm, Sweden: Carlsson bokförlag; 2006. 272 p.
2. Nordlöf B. Svenska adoptioner i Stockholm 1918-1973 Stockholm, Sweden: Socialtjänstförvaltningen, Forsknings- och utvecklingsenheten; 2001. 107 p.
3. Bohman M. Study of Adopted Children, Their Background, Environment and Adjustment. Acta Paediatr Scand. 1972;61(1):90-&.
4. Vinnerljung B, Hjern A. Cognitive, educational and self-support outcomes of long-term foster care versus adoption. A Swedish national cohort study. Child Youth Serv Rev. 2011;33(10):1902-10.
5. Data from the Population and Housing Censuses (FoB) [Available from: https://www.scb.se/vara-tjanster/.
6. Ludvigsson JF, Almqvist C, Bonamy AK, Ljung R, Michaëlsson K, Neovius M, et al. Registers of the Swedish total population and their use in medical research. Eur J Epidemiol. 2016;31(2):125-36.
7. Ekbom A. The Swedish Multi-generation Register. Methods Mol Biol. 2011;675:215-20.
8. Socialstyrelsen. The National Patient Register [Available from: https://www.socialstyrelsen.se/en/statistics-and-data/registers/national-patient-register/.
9. Ludvigsson JF, Andersson E, Ekbom A, Feychting M, Kim JL, Reuterwall C, et al. External review and validation of the Swedish national inpatient register. BMC Public Health. 2011;11:450.
10. Frisell T, Lichtenstein P, Långström N. Violent crime runs in families: a total population study of 12.5 million individuals. Psychol Med. 2011;41(1):97-105.
11. Brooke HL, Talbäck M, Hörnblad J, Johansson LA, Ludvigsson JF, Druid H, et al. The Swedish cause of death register. Eur J Epidemiol. 2017;32(9):765-73.
12. Ludvigsson JF, Svedberg P, Olén O, Bruze G, Neovius M. The longitudinal integrated database for health insurance and labour market studies (LISA) and its use in medical research. Eur J Epidemiol. 2019;34(4):423-37.
13. Ludvigsson JF, Berglind D, Sundquist K, Sundström J, Tynelius P, Neovius M. The Swedish military conscription register: opportunities for its use in medical research. Eur J Epidemiol. 2022;37(7):767-77.
14. Lichtenstein P, De Faire U, Floderus B, Svartengren M, Svedberg P, Pedersen NL. The Swedish Twin Registry: a unique resource for clinical, epidemiological and genetic studies. J Intern Med. 2002;252(3):184-205.
15. Welfare) STNBoHa. National Medical Birth Register [Available from: https://www.socialstyrelsen.se/en/statistics-and-data/registers/national-medical-birth-register/.
16. Black S, Grönqvist E, Öckert B. Born to Lead? The Effect of Birth Order on Non-Cognitive Abilities. The Review of Economics and Statistics. 2017;100.
17. Cesarini D, Johannesson M, Magnusson P, Wallace B. The Behavioral Genetics of Behavioral Anomalies. Management Science. 2012;58:21-34.
18. Lilieblad B, Ståhlberg B. Reliabilitet hos psykologiska bedömningar vid inskrivnings prövning. Stockholm: Försvarets forskningsanstalt (FOA); 1977 1977/12.

19. Pettersson E, Larsson H, D'Onofrio BM, Bölte S, Lichtenstein P. The general factor of psychopathology: a comparison with the general factor of intelligence with respect to magnitude and predictive validity. World Psychiatry. 2020;19(2):206-13.
